# Prevalence and Characteristics of Glaucoma Among Patients Presenting to Ophthalmology Clinics in a Tertiary Hospital in the Kingdom of Bahrain

**DOI:** 10.7759/cureus.54129

**Published:** 2024-02-13

**Authors:** Khatoon A Husain, Haneen Alaali, Ghadeer G Alarayedh

**Affiliations:** 1 Ophthalmology Department, Salmaniya Medical Complex, Manama, BHR; 2 Ophthalmology Department, Al Arrayed Eye Center, Manama, BHR

**Keywords:** bahrain, blindness, humans, visual impairment, glaucoma, prevention of blindness, neovascular glaucoma, chronic angle closure glaucoma, normal- tension glaucoma, primary open angle glaucoma

## Abstract

Introduction

The aim of this study was to determine the prevalence and characteristics of glaucoma in patients presenting to the ophthalmology department in a tertiary hospital in Bahrain for the first time.

Methods

A retrospective study was conducted at the Salmaniya Medical Complex, Manama, Bahrain. The medical records of all patients who presented for the first time to an eye clinic between January and December 2019 were reviewed. Patients who were diagnosed with glaucoma were included in this study. Data regarding age, sex, ethnicity, type of glaucoma, previous treatment, best corrected visual acuity, cup-to-disc ratio, intraocular pressure, central corneal thickness, optical coherence tomography (OCT) retinal nerve fiber layer (RNFL), and visual field findings were collected.

Results

Of a total of 18,238 new patients in 2019, 173 patients (0.97%) had glaucoma. The mean age of patients with glaucoma was 59.6 ± 11.3 years and approximately 60% of them were males (n=103, 59.5%). In 93% of the cases, glaucoma involved both eyes (n=161). Primary open-angle glaucoma (n=97, 56.1%), normal tension glaucoma (n=28, 16.2%), and chronic angle closure glaucoma (n=15, 8.7%) were the most frequently encountered types of glaucoma. Approximately 16.76% (n=29) of the patients were blind in one or two eyes at the time of presentation.

Conclusion

There seems to be a low prevalence of glaucoma among the encountered cases on the first visit to ophthalmology clinics in Bahrain, with primary open-angle glaucoma being the most common type.

## Introduction

Glaucoma, defined as optic nerve damage that is accompanied by characteristic atrophy, cupping, and visual field defects, is a cause of irreversible blindness globally [1,2]. In fact, it is the leading cause of blindness in the world after cataracts. It affects as many as 80 million patients in the world and results in bilateral blindness in around 10% of them [3].

The exact pathogenesis of glaucoma remains unclear. However, glaucoma is a multifactorial disease with involvements of vascular, genetic, anatomical, and immune elements in its pathogenesis[4].

There are different types of glaucoma; the main two types are open-angle glaucoma and angle-closure glaucoma. Open-angle glaucoma is the most frequent subtype and presents in more than 80% of glaucoma cases, while angle-closure glaucoma presents suddenly with a higher risk of visual loss. In addition, there are secondary causes of glaucoma including inflammation, tumor, and medications like corticosteroids[5].

Many patients with glaucoma remain asymptomatic until it advances. Clinical presentations of glaucoma are variable and range from incidental detection during an ophthalmic examination to mild symptoms like loss of visual acuity, pain, conjunctival erythema, and corneal oedema to blindness. It takes around 25 years for untreated patients to develop visual loss[6].

Early detection and treatment can prevent ocular damage in patients with glaucoma; however, mass population screening is not recommended. Nonetheless, all patients presenting for eye care should be reviewed for glaucoma risk factors and undergo clinical examination to rule out glaucoma. In addition, the World Health Organization recommends adopting a public health approach to address avoidable blindness due to primary glaucoma [7].

Several studies were conducted to assess the epidemiology and sub-types of glaucoma which showed high global variations. A systematic review concluded that the prevalence of glaucoma across the world is 3.54% in people aged 40-80 years, with primary open-angle glaucoma being highest in Africa (4.20%), and primary angle-closure glaucoma being highest in Asia (1.09%) [8]. Another recent systematic review revealed a global prevalence of 0.6% for primary angle-closure glaucoma and a higher prevalence was reported among females (RR= 0.71) and Asians [9]. One study done across Asia revealed a prevalence of 3.4-3.7% across Asia with primary open-angle glaucoma being the most prevalent (2.34%; 95% CI 0.96-4.55), followed by primary angle-closure glaucoma (0.73%; 95% CI 0.18-1.96) and secondary glaucoma (0.47%; 95%CI 0.09-1.48) [10]. In Iran, the prevalence of glaucoma was found to be 4.4%, with most being asymptomatic (89.7%). Primary open-angle glaucoma (3.2%) and primary angle-closure glaucoma (0.4%) were the most prevalent types [11].

In Gulf Corporation Council (GCC) countries, several studies assessed the prevalence and characteristics of patients with glaucoma. A study of 940 people in Saudi Arabia found a prevalence of 5.6%, with a higher prevalence among males with most having mild-moderate symptoms (73%) and less than 1% having bilateral blindness (0.8%) [12]. In Qatar, a study of 3149 participants reported a glaucoma prevalence of 1.73%, with open-angle glaucoma (65.7%) being the most frequent type, followed by other glaucoma, like post-traumatic, pseudo-exfoliation (20.9%) and angle-closure glaucoma (13.4%)[13].

A study conducted in Saudi Arabia and recruited 999 participants with glaucoma found that 82.3% of them had bilateral involvement, and primary open-angle glaucoma (27.7%) and secondary glaucoma (26.7%) were the most prevalent types[14].

The present study aimed to determine the prevalence and characteristics of glaucoma among all encountered cases in a tertiary care centre in Bahrain. This will help in guiding the designs of glaucoma screening programs for early detection and treatment.

## Materials and methods

Study design and setting

A cross-sectional study was conducted in Salmaniya Medical Complex between January 1st and December 31st, 2019. Salmaniya Medical Complex (SMC) is the largest tertiary hospital in Bahrain and is considered the main governmental healthcare facility serving a major percentage of the population in the Kingdom. It provides primary eye care for patients and is the referral centre for eye healthcare providers.

Approval was obtained from the Secondary Health Research Subcommittee to review patients' records in SMC. The study adhered to the Declaration of Helsinki guidelines. 

Selection criteria and process

All patients who attended outpatient ophthalmology clinics for the first time were assessed for eligibility. All patients who had glaucoma confirmed by glaucoma specialist examination were included. Glaucoma patients diagnosed and followed in the glaucoma clinic before 2019 were excluded from the study.

Selection process

Data including age, sex, family history, associated medical illness, type of glaucoma, previous glaucoma diagnosis and treatment, corrected Snellen’s visual acuity, slit lamp examination findings, including intraocular pressure measured by applanation tonometer, anterior segment, retina, and gonioscopy findings were retrieved from the electronic medical records. In addition to the visual field, optical coherence tomography (OCT) retinal nerve fiber layer (RNFL), and central corneal thickness results were also extracted.

Definitions

Glaucoma was classified according to the International Society of Geographical and Epidemiological Ophthalmology (ISGEO) criteria. Primary open-angle glaucoma (POAG) was characterized by glaucomatous optic neuropathy in the presence of an open angle and no other ocular abnormality to account for a secondary mechanism, while primary angle-closure glaucoma (PACG) was characterized by the presence of primary angle closure plus glaucomatous optic neuropathy. The diagnosis of secondary glaucoma was based mainly upon the presence of high intraocular pressure, evident disc damage, and ocular pathological processes such as neovascularization, pseudo-exfoliation, pigment dispersion, lens pathology, uveitis, trauma, and surgical complications [15]. Patients were considered to have blindness if the visual acuity was worse than 0.05 (3/60), or if they had a visual field less than 10° around central fixation [16].

Statistical analysis

Data analyses were carried out using SPSS 26 software (IBM Corp., Armonk, USA). Quantitative variables were summarised using means and standard deviations (SD) while categorical variables were summarised using frequencies and percentages. Bar charts and pie charts were used to present categorical variables. Mann-Whitney test was used to compare the mean between two groups while the Kruskal-Wallis test was used to compare the mean between more than two groups. A p-value of less than 0.05 was considered statistically significant.

## Results

Out of 18,238 encountered patients, 173 had glaucoma, with an average age of 59.6 ± 11.3 years. The prevalence of glaucoma patients among first-visit patients attending eye clinics in 2019 is (0.97%, 173/18,238). More than 70% of the cohort was aged 50-70 years (n=126) and approximately 60% of them were males (n=103, 59.5%). Table 1 presents the demographic characteristics of the glaucoma patients.

**Table 1:** Demographic characteristics of glaucoma patients (n = 173) *The mean age is 59.6 ± 11.3 years.

Demographic characteristics	n (%)
Age*	
<50 years	25 (14.5)
50 - 70 years	126 (72.8)
>70 years	22 (12.7)
Gender	
Male	103 (59.5)
Female	70 (40.5)
Nationality	
Bahraini	162 (93.6)
Non-Bahraini	11 (6.4)

In most participants, glaucoma affected both eyes (n=161; 93.1%). The average intraocular pressure was 22.2 ± 9.2 mmHg in the right eye and slightly higher in the left eye (22.8 ± 10.5 mmHg). Primary open-angle glaucoma (n=97, 56.1%), normal tension glaucoma (n=28, 16.2%), and chronic angle closure glaucoma (n=15, 8.7%) were the most frequently encountered types of glaucoma (Table 2).

**Table 2:** Glaucoma and eye characteristics of the patients OD: right eye; OS: left eye

Glaucoma and eye characteristics	n (%)
Eye side	
Bilateral	161 (93.1)
Right	8 (4.6)
Left	4 (2.3)
Intraocular pressure	
OD, mean SD	22.2 ± 9.2
OS, mean SD	22.8 ± 10.5
Central corneal thickness	
OD, mean SD	520 ± 44.4
OS, mean SD	521 ± 44.9
Type of glaucoma	
Primary open-angle glaucoma	97 (56.1)
Normal tension glaucoma	28 (16.2)
Chronic angle-closure glaucoma	15 (8.7)
Neovascular glaucoma	11 (6.4)
Acute angle-closure glaucoma	6 (3.5)
Congenital glaucoma	1 (0.6)
Other secondary glaucoma	15 (8.7)

As shown in Table 3, patients with acute angle-closure glaucoma were older (66.0 ± 11.3 years) compared to patients with other types of glaucoma.

**Table 3:** The mean age in years of types of glaucoma

Type of glaucoma	Age in years (mean ± SD)
Primary open-angle glaucoma	60.2 ± 9.9
Neovascular glaucoma	58.3 ± 15.3
Normal tension glaucoma	57.7 ± 8.2
Chronic angle-closure glaucoma	59.6 ± 9.9
Acute angle-closure glaucoma	66.0 ± 11.3
Congenital glaucoma	2.0
Other secondary glaucoma	62.1 ± 13.9

Table 4 presents the best corrected visual acuity of glaucoma patients. More than half of the patients had a normal corrected visual acuity in both eyes (51.2% and 53.2% for right and left eyes, respectively).

**Table 4:** Best corrected visual acuity of glaucoma patients ^a^ Number of missing values = 7; ^b ^Number of missing values = 7

Best Corrected Visual Acuity	Right eye n (%)	Left eye n (%)
6/6	83 (51.2)	84 (53.2)
6/9	22 (13.6)	28 (17.7)
6/12	10 (6.2)	14 (8.9)
6/18	17 (10.5)	3 (1.9)
6/24	9 (5.6)	10 (6.3)
6/36	2 (1.2)	6 (3.8)
6/60	5 (3.1)	6 (3.8)
Counting fingers	8 (4.9)	4 (2.5)
No perception of light	4 (2.5)	1 (0.6)
Hand motion	2 (1.2)	2 (1.3)
Total	162^a^ (100)	158^b^ (100)

**Figure 1:**
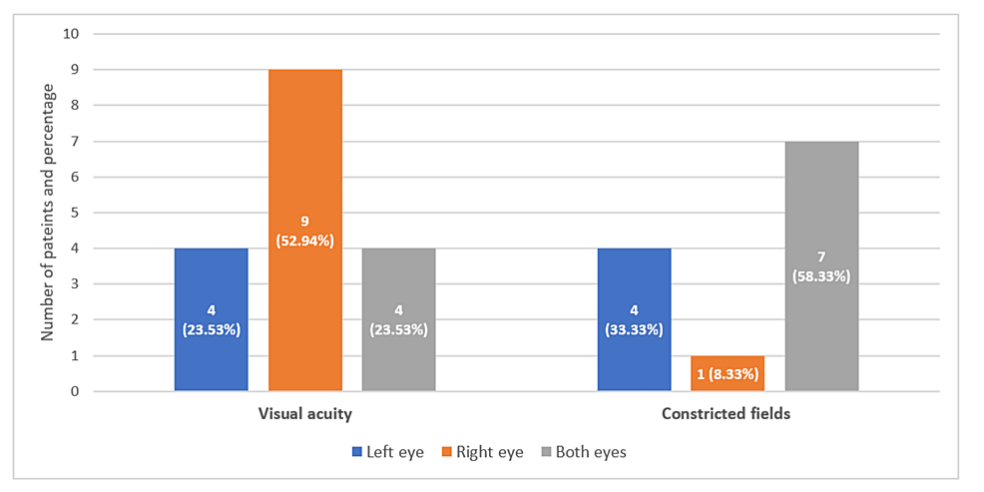
Prevalence of blindness among patients with glaucoma

As shown in Figure 1, the prevalence of blindness among the cohort was 16.76% (n=29/173). Specifically, 17 patients (9.83%) had visual acuity defects while 12 patients (6.94%) had constricted fields.

No significant differences between demographic characteristics and intraocular pressure were found except for non-Bahrainis, who had a higher left-eye pressure compared to Bahrainis (p=0.019) (Table 5). The association between demographic characteristics and the central corneal thickness assessed is presented in Table 6. Bahraini patients had a higher central corneal thickness in the left eye compared to non-Bahraini (p=0.013).

**Table 5:** Association between demographic characteristics and intraocular pressure ^a^ Kruskal-Wallis test; ^b^ Mann-Whitney test; IOP: intraocular pressure

Demographic characteristics	IOP Right eye (Mean ± SD)	P-value	IOP Left eye (Mean ± SD)	P-value
Age				
<50 years	23.5 ± 11.5	0.284	24.7 ± 12.5	0.591^a^
50 - 70 years	21.5 ± 8.5		22.4 ± 10.5	
>70 years	25 ± 9.9		23.1 ± 8	
Gender				
Male	23 ± 9.9	0.204	24 ± 11.8	0.274^b^
Female	20.9 ± 7.9		21.3 ± 8.2	
Nationality				
Bahraini	22.1 ± 9	0.989	22.1 ± 9.7	0.019^b^
Non-Bahraini	23.6 ± 11.7		34.3 ± 15.9	

**Table 6:** Association between demographic characteristics and Central Corneal Thickness

	Right eye	P-value	Left eye	P-value
	Mean ± SD (μm)		Mean ± SD (μm)	
Age				
<50 years	500.4 ± 44.1	0.230	492.9 ± 40.4	0.050^a^
50 - 70 years	524.1 ± 45.1		525.4 ± 45.2	
>70 years	510.3 ± 33.1		523.1 ± 33.4	
Gender				
Male	520.7 ± 41.5	0.624	524 ± 41.4	0.250^b^
Female	519.2 ± 48.2		517.4 ± 48.9	
Nationality				
Bahraini	521.4 ± 44.1	0.085	523.3 ± 44.2	0.013^b^
Non-Bahraini	488.4 ± 45.2		479 ± 38.1	
a. Kruskal-Wallis test. b. Mann-Whitney test.				

As shown in Table 7, all types of glaucoma occurred mainly in patients aged 50-70 years except for congenital glaucoma. Neovascular glaucoma occurred solely in males (n=11, 10.7%).

**Table 7:** Distribution of types of glaucoma according to age, gender, and nationality

Type of glaucoma	Age in years			Gender		Nationality	
	<50	50 - 70	>70	Male	Female	Bahraini	Non-Bahraini
	n (%)	n (%)	n (%)	n (%)	n (%)	n (%)	n (%)
Primary open-angle glaucoma	10 (40)	75 (59.5)	12 (54.5)	54 (52.4)	43 (61.4)	96 (59.3)	1 (9.1)
Neovascular glaucoma	4 (16)	6 (4.8)	1 (4.5)	11 (10.7)	0 (0)	10 (6.2)	1 (9.1)
Normal tension glaucoma	4 (16)	23 (18.3)	1 (4.5)	11 (10.7)	17 (24.3)	25 (15.4)	3 (27.3)
Chronic angle-closure glaucoma	2 (8)	12 (9.5)	1 (4.5)	11 (10.7)	4 (5.7)	12 (7.4)	3 (27.3)
Acute angle closure glaucoma	1 (4)	2 (1.6)	3 (13.6)	3 (2.9)	3 (4.3)	4 (2.5)	2 (18.2)
Congenital glaucoma	1 (4)	0 (0)	0 (0)	1 (1)	0 (0)	1 (0.6)	0 (0)
Other secondary glaucoma	3 (12)	8 (6.3)	4 (18.2)	12 (11.7)	3 (4.3)	14 (8.6)	1 (9.1)
Total	25 (100)	126 (100)	22 (100)	103 (100)	70 (100)	162 (100)	11 (100)

## Discussion

The present study aimed to determine the prevalence and characteristics of glaucoma among patients presenting to ophthalmology clinics in a tertiary hospital in the Kingdom of Bahrain. The results revealed a low prevalence of glaucoma among the encountered cases. Glaucoma was more prevalent among males and involved both eyes. In addition, primary open-angle and normal tension glaucoma were the most prevalent types.

The prevalence of glaucoma varies across the studies. Here, a prevalence of less than 1% was found. Several studies reported a higher prevalence of glaucoma. For instance, studies in Saudi Arabia, Oman, Qatar, and Iran reported a higher prevalence of glaucoma. This variation in glaucoma prevalence can be due to differences in the definitions of glaucoma, different settings, different diagnostic tools, and different genetic factors of patients. Many studies assessed the prevalence of glaucoma among the general population, which might affect the prevalence of glaucoma.

The findings of the present study are in line with the reported figures about the most prevalent type of glaucoma. Here, primary open-angle glaucoma was the most encountered type. Similarly, many studies in Middle Eastern countries like Qatar, Saudi Arabia, and Iran, and in Asia and America reported the same findings [10-14].

Most patients in this study had bilateral involvement. This finding is expected as the pathogenesis of glaucoma affects both eyes similarly. The same findings were reported by Helayel et al. [14]. Some studies revealed bilateral involvement only with specific types of glaucoma like primary open-angle glaucoma[17,18].

The sex differences in the prevalence of glaucoma were assessed as well in the literature. Here, most patients with glaucoma were male. While some studies found a male predominance in glaucoma, other studies showed the opposite [19,20]. Some studies reported higher rates of acute closure glaucoma among females and higher rates of primary open-angle glaucoma among females [19,20].

Approximately one in every six patients (16.76%) was legally blind in at least one eye at the time of presentation in this study. Similar rates were reported in the literature. Quigley and Broman estimated the prevalence of legal blindness among glaucomatous patients to be around 13.9% [3]. A higher prevalence of legal blindness was reported by Al-Najmi et al (26.6%)[21].

This study has several strengths. It is the first study to determine the prevalence as well as the characteristics of glaucoma in Bahrain. All patients who visited the ophthalmology clinic for the first time were included in the present study. In addition, several factors regarding the characteristics, types, and severity of glaucoma were assessed. However, there are some limitations as well. Participants’ comorbidities such as diabetes mellitus, hypertension, and dyslipidemia were not assessed. Medication history was not assessed as well. These factors might be related to glaucoma and could be involved in its pathogenesis.

## Conclusions

In conclusion, this study revealed a low prevalence of glaucoma among the encountered cases on the first visit to the ophthalmology clinic, with primary open-angle glaucoma being the most prevalent type. Since one in six patients had unilateral or bilateral legal blindness, early detection and management of patients with glaucoma is essential to prevent glaucoma-related complications such as blindness.
